# Molecular and phenotypic characterization of clinical isolates belonging to a KPC-2-producing strain of ST15 *Klebsiella pneumoniae* from a Vietnamese pediatric hospital

**DOI:** 10.1186/s13756-019-0613-4

**Published:** 2019-10-16

**Authors:** Björn Berglund, Ngoc Thi Bich Hoang, Maria Tärnberg, Ngai Kien Le, Maud Nilsson, Dung Thi Khanh Khu, Olov Svartström, Jenny Welander, Lennart E. Nilsson, Linus Olson, Tran Minh Dien, Hai Thanh Le, Mattias Larsson, Håkan Hanberger

**Affiliations:** 1 Department of Clinical and Experimental Medicine, Linköping University, Linköping, Sweden; 2 Vietnam National Children’s Hospital, Hanoi, Vietnam; 3 Training and Research Academic Collaboration (TRAC)– Sweden – Vietnam, Hanoi, Vietnam; 4 Department of Clinical Microbiology and Department of Clinical and Experimental Medicine, Linköping University, Linköping, Sweden; 5 Karolinska Institutet, Stockholm, Sweden

**Keywords:** Antibiotic resistance, Carbapenems, *Klebsiella pneumoniae*, Vietnam, Whole-genome sequencing

## Abstract

**Background:**

Carbapenem-resistant *Klebsiella pneumoniae* are becoming increasingly common in hospital settings worldwide and are a source of increased morbidity, mortality and health care costs. The global epidemiology of carbapenem-resistant *K. pneumoniae* is characterized by different strains distributed geographically, with the strain ST258 being predominant in Europe and USA, and ST11 being most common in East Asia. ST15 is a less frequently occurring strain but has nevertheless been reported worldwide as a source of hospital outbreaks of carbapenem-resistant *K. pneumoniae*.

**Methods:**

In this study, whole-genome sequencing and antimicrobial susceptibility testing was used to characterize 57 clinical isolates of carbapenem-resistant *K. pneumoniae* belonging to a strain of ST15, which were collected at a Vietnamese pediatric hospital from February throughout September 2015.

**Results:**

Aside from the carbapenem resistance gene *bla*_KPC-2_, which was carried by all isolates, prevalence of resistance genes to other antibiotics including aminoglycosides, macrolides, quinolones, fosfomycin and trimethoprim, was also high. All isolates were multidrug-resistant. Susceptibility was highest to ceftazidime/avibactam (96%), gentamicin (91%) and tigecycline (82%). Notably, the colistin resistance rate was very high (42%). Single-nucleotide polymorphism analysis indicated that most isolates belonged to a single clone.

**Conclusions:**

The diverse variety of antibiotic resistance genes and the high antibiotic resistance rates to last-resort antibiotics such as carbapenems and colistin, is indicative of a highly adaptable strain. This emphasizes the importance of implementation of infection controls measures, continued monitoring of antibiotic resistance and prudent use of antibiotics to prevent further selection of resistant strains and the emergence of pan-resistant clones.

## Introduction

Emerging multidrug-resistant gram-negative bacteria are a global problem causing increased morbidity, mortality and health care costs, which threaten the modern medical system. Particularly problematic is the dissemination of genes encoding carbapenem resistance among *Klebsiella pneumoniae*, with increasing prevalence reported in Europe [1], USA [2] and China [3]. The global epidemiology of carbapenem-resistant *K. pneumoniae* (CR-KP) is characterized by the spread of strains with mainly KPC-, NDM- and OXA-48-carbapenemases, and the distribution of strains differ geographically. The ST258 clonal group is dominating in Europe and USA whereas ST11 is more common in East Asian countries. ST15 is another *K. pneumoniae* clonal group associated with production of extended-spectrum β-lactamases (ESBLs) and carbapenemases and has been indicated in clinical cases and hospital outbreaks worldwide [4]. Examples include isolates with KPC-2 in Bulgaria [5], KPC-3-producers in Portugal [6], OXA-48-producing isolates in Spain [7] and Vietnam [8], isolates with OXA-232 in China [9] and NDM-1-producing isolates in Nepal [10]. While ST15 isolates in specific outbreaks can be highly homogenic [9, 11], a high variability in antibiotic susceptibility and antibiotic resistance determinants among ST15 isolates from different study locations are indicative of a group of bacteria with high potential for horizontal gene acquisition.

In 2012 and 2013 we conducted monthly point prevalence surveys at intensive care units (ICUs) in 16 Vietnamese hospitals. The results in the adult ICUs, with 3287 cases, showed a hospital-acquired infection prevalence of 29.5%, most frequently caused by *Acinetobacter baumannii*, *Pseudomonas aeruginosa*, and *K. pneumoniae*, with carbapenem resistance rates of 89.2, 55.7, and 14.9% respectively [12]. In the ICUs at three pediatric referral hospitals, 1363 cases were surveyed. Hospital-acquired infections were diagnosed in one third of the cases, most commonly caused by *K. pneumoniae*, among which the carbapenem resistance rate was 55% [13]. This initiated further investigation of the epidemiology of CR-KP isolated from patients at one of the abovementioned hospitals during 2015 using whole-genome sequencing (WGS). Screening of all isolates collected during 2015 for carbapenem- and colistin-resistant isolates of *K. pneumoniae* revealed that all isolates (*n* = 31) with this antibiotic resistance phenotype belonged to a genetically homogenous strain of ST15 showing a high heterogeneity in mutations engendering colistin resistance [14]. The possibility of a hospital outbreak caused by this ST15 strain prompted further screening for ST15 among carbapenem-resistant but colistin-susceptible isolates. The aim with this study was to elucidate the epidemiology, antimicrobial susceptibility and molecular characteristics of a ST15 strain of CR-KP possibly responsible for a clonal outbreak at a large Vietnamese pediatric hospital.

## Materials and methods

### Clinical isolates and patient data

This study was carried out at a large pediatric hospital in Vietnam. From February throughout September 2015, all clinical isolates of *K. pneumoniae* obtained from diagnostic routine at the hospital were tested for resistance to imipenem and meropenem with antimicrobial susceptibility testing by using the VITEK 2 system (BioMérieux, Marcy-l‘Étoile, France). WGS was carried out among imipenem- and meropenem-resistant isolates to elucidate the dissemination of a CR-KP ST15 strain among the patients at the hospital. In addition to 22 isolates of colistin-resistant CR-KP ST15 collected from 22 patients during this period for a previous study [14], 35 unique isolates of ST15 CR-KP were further determined in the current study. A total of 57 unique isolates from 57 unique patients were thus included for further study. Partial background information collected included dates of admission and discharge, date of birth, whether the patient was originally hospitalized at the study hospital or transferred from another hospital, diagnosis, outcome, date of culture, intubation, sex, specimen, and department at which the patient was interred.

### DNA extraction, library preparation and whole-genome sequencing

DNA was extracted using the EZ1 DNA Tissue Kit and the EZ1 Advanced XL instrument (Qiagen, Hilden, Germany). The extracted DNA was stored in − 20 °C before subsequent use in library preparation. A sequencing library was constructed using Nextera XT kit (Illumina, San Diego, CA). As input DNA, 1 ng of genomic DNA was used. The DNA concentration, determined by a Qubit 2.0 Fluorometer (Thermo Fisher Scientific, Waltham, MA), was adjusted to 0.2 ng/μl in RNAse-free water. Sequencing was performed on a MiSeq instrument (Illumina), and produced a mean sequencing depth of 32x.

### Genome assembly and bioinformatic analysis

Genome assembly and subsequent bioinformatic analyses were performed with CLC Genomics Workbench v.9.5.3 (Qiagen). Raw reads were uploaded to the Sequence Read Archive at NCBI (Accession numbers: SRR6656601-SRR6656635). Determination of antibiotic resistance genes and multilocus sequence typing was performed by querying the databases at the Center for Genomic Epidemiology (http://www.genomicepidemiology.org/). The virulence gene querying and capsule typing via the *wzi* gene was performed using the Institut Pasteur MLST and whole genome MLST databases (http://bigsdb.pasteur.fr/). Variants were called against a reference genome (NC_022566) if they had a sequencing depth of ≥10x, a frequency of ≥90% and a Phred score ≥ 20, and the resulting variants were used to determine the genetic relationship between the isolates using the single nucleotide polymorphism (SNP) analysis tool in CLC Genomics Workbench. Criteria for including SNPs in the phylogenetic tree were set to a sequencing depth of ≥10x, a distance between SNPs of ≥10 bp and a Z-score ≥ 1.96. Due to low average sequencing depth (<15x), samples VN26 and VN918 were excluded from the analysis.

### Antimicrobial susceptibility testing

Carbapenem resistance for the CR-KP ST15 isolates were confirmed by determining MICs of meropenem by using Etests (BioMérieux) on Muller-Hinton agar (Oxoid, Basingstoke, England). Furthermore, Etests were used to determine MICs for the antibiotics ertapenem, imipenem, amikacin, gentamicin, tobramycin, fosfomycin, chloramphenicol, trimethoprim/sulfamethoxazole, ciprofloxacin and tigecycline. MICs of colistin were determined by using the broth microdilution method with cation-adjusted BBL Mueller-Hinton broth (BD, Franklin Lakes, NJ, USA) and Sensititre plates (Thermofisher Scientific, Waltham, MA, USA). Antimicrobial susceptibility was determined using the clinical breakpoints from the European Committee for Antimicrobial Susceptibility Testing (EUCAST, 2017). Isolates were considered multidrug-resistant if non-susceptible to antibiotics in three or more groups of antibiotics [15].

## Results

### Patient data

Partial patient data were collected from a total of 57 unique patients from which the 57 analyzed isolates originated. 28% (*n* = 16) of the patients were female. The median age based on data available from 56 (98%) patients was 36.0 days. The average duration of hospital stay based on available data from 49 (86%) patients was 44.6 days. The baseline data on the patients from which the samples were collected are presented in Table 1. Data on patient outcome was available for 29 (51%) of the patients included in the study. The mortality among these patients was 14%. Among the 27 patients with missing outcome data, 19 were withdrawn from treatment due to pessimal prognosis with no further treatment option. Data on patient origin (community or transfer from other hospital) was available for 41 (72%) patients, 22% of whom originated from the community whereas 78% had been transferred from other hospitals. Data on patient diagnoses were available for 53 (93%) patients. The most common diagnoses among these patients were respiratory failure (32%), pneumonia (28%) and cardiovascular disease (15%). A total of four patients (7%) were diagnosed with septicemia, one of which had an isolate of *K. pneumoniae* isolated from their blood. The samples from which the isolates were collected were tracheal fluid (61%), nasopharynx (23%), blood (5%) and other specimens (11%).

**Table 1:** Baseline parameters for the 57 patients at a large Vietnamese pediatric hospital included in the study, in relation to treatment outcome, from which carbapenem-resistant ST15 *K. pneumoniae* were isolated

	Total	Discharged	Death	N.A.^a^
Sex				
Female	16	9	0	7
Male	41	16	4	21
Diagnosis				
Respiratory failure	17	7	2	8
Pneumonia	15	8	1	6
Cardiovascular disease	8	4	2	2
Septicemia	5	2	2	1
Pre-term	5	2	2	1
Enterocolitis/peritonitis	3	2	0	1
Esophagus atresia	3	1	0	2
Cerebral hemorrhage	2	1	0	1
Diaphragmatic hernia	2	0	0	2
Other	7	3	0	4
N.A.	4	0	0	4
Origin				
Other hospital	32	17	1	14
Community	9	3	2	4
N.A.	16	5	1	10
Department				
General ICU	8	3	0	5
Neonatal ICU	28	16	1	11
Surgical ICU	17	5	3	9
Other	4	1	0	3
Invasive procedure				
Intubation	45	18	4	23
Central vascular catheter	36	13	4	19
Urinary catheter	20	6	2	12
N.A.	4	0	0	4
Specimen				
Tracheal fluid	35	13	3	19
Nasopharynx	12	7	0	5
Blood	3	1	0	2
Other	6	4	1	1

*ICU* intensive care unit

^a^ Data not available

### Antibiotic resistance genes, virulence genes and *wzi*-typing

Data on antibiotic resistance genes are presented in Table 2. All isolates carried the carbapenemase gene *bla*_KPC-2_, however, two isolates also carried *bla*_NDM-1_. Carriage rates of beta-lactamase gene *bla*_OXA-9_, aminoglycoside resistance genes *aac (6′)-*Ib and *aadA1*, fosfomycin resistance gene *fosA,* trimethoprim resistance gene *dfrA23* and quinolone resistance genes *oqxA* and *oqxB* were also high (> 90%). The ribosomal methylase gene *rmtB*, which confer high-grade resistance to aminoglycosides, was detected in five isolates (9%). All isolates carried the *mrk* operon *mrkABCDFJ* coding for type 3 fimbriae, with four isolates additionally carrying the chromosomal *mrk* operon transcriptional activator genes *mrkI* and *mrkH*. The aerobactin siderophore-encoding operon *iucABCD*-*iutA* was detected in 48 out of the 57 isolates (84%). All isolates carried the iron uptake system encoded by the *kfuA*, *kfuB* and *kfuC* genes. *rmpA2*, a gene encoding a transcriptional activator for capsular polysaccharide synthesis, was detected in one isolate, VN956B. Because of an insertion causing a frameshift, the predicted gene product of *rmpA2* in VN956B was only 73 aa long as compared to the 212 aa long wild type protein. This mutation likely resulted in a dysfunctional gene product. The capsular serotype as determined through *wzi*-typing, was determined to be K10 for all isolates except isolates VN915 and VN918, for which no serotype could be determined.

**Table 2:** Genotypic features of 57 carbapenem-resistant ST15 *K. pneumoniae* isolated at a large Vietnamese hospital. Percentages are denoted in parenthesis

Genes	Rate	Function	Genes	Rate	Function
*bla*_KPC-2_	57 (100)	β-lactam resistance	*mph(A)*	39 (68)	Macrolide resistance
*bla*_NDM-1_	2 (4)	β-lactam resistance	*aac(6′)-*Ib	52 (91)	Aminoglycoside resistance
*bla*_SHV-12_	36 (63)	β-lactam resistance	*aadA1*	52 (91)	Aminoglycoside resistance
*bla*_SHV-28_	48 (84)	β-lactam resistance	*aph(3″)-*Ib	8 (14)	Aminoglycoside resistance
*bla*_SHV-73_	6 (11)	β-lactam resistance	*aph(6)-*Id	8 (14)	Aminoglycoside resistance
*bla*_SHV-155_	1 (2)	β-lactam resistance	*rmtB*	5 (9)	Aminoglycoside resistance
*bla*_TEM-1B_	16 (28)	β-lactam resistance	*sul1*	8 (14)	Sulphonamide resistance
*bla*_TEM-199_	36 (63)	β-lactam resistance	*sul2*	8 (14)	Sulphonamide resistance
*bla*_OXA-9_	52 (91)	β-lactam resistance	*dfrA16*	8 (14)	Trimethoprim resistance
*fosA*	57 (100)	Fosfomycin resistance	*dfrA23*	56 (98)	Trimethoprim resistance
*fosA3*	5 (9)	Fosfomycin resistance	*iucABCD-iutA*	48 (84)	Iron-acquisition
*oqxA*	57 (100)	Quinolone resistance	*mrk* operon	57 (100)	Adhesion
*oqxB*	57 (100)	Quinolone resistance	*kfuABC*	57 (100)	Iron-uptake
*cmlA1*	8 (14)	Phenicol resistance	*rmpA2*	1 (2)	Regulator of mucoid phenotype

### Antimicrobial susceptibility testing

Data on antimicrobial susceptibility, MIC_50_ and MIC_90_ values are presented in Table 3. All isolates were multidrug-resistant. Susceptibility was highest to the antibiotics ceftazidime/avibactam (96%), gentamicin (91%), tigecycline (82%), trimethoprim/sulfamethoxazole (70%) and fosfomycin (70%). Susceptibility was low to colistin (58%) and chloramphenicol (3.5%), and 0% for the other tested antibiotics.

**Table 3:** Antimicrobial susceptibility pattern of 57 isolates of carbapenem-resistant ST15 *K. pneumoniae* isolated at a large Vietnamese pediatric hospital

Antimicrobial agent	S ≤ (mg/L)	R > (mg/L)	S (%)	R (%)	MIC_50_ (mg/L)	MIC_90_ (mg/L)
Gentamicin	2	4	91	8.8	1.5	2
Tobramycin	2	4	0	100	16	24
Amikacin	8	16	0	96	32	64
Chloramphenicol	8	8	3.5	96	128	> 256
Trimethoprim/sulfamethoxazole	2	4	70	25	2	> 32
Ciprofloxacin	0.25	0.5	0	100	> 32	> 32
Cefotaxime	1	2	0	100	> 32	> 32
Imipenem	2	8	0	96	> 32	> 32
Meropenem	2	8	0	100	> 32	> 32
Ertapenem	0.5	1	0	100	> 32	> 32
Tigecycline	1	2	82	7.0	0.5	2
Fosfomycin	32	32	70	30	16	> 1024
Ceftazidime/avibactam	8	8	96	3.5	2	3
Colistin	2	2	58	42	0.5	32

### Single-nucleotide polymorphism analysis

**Fig. 1:**
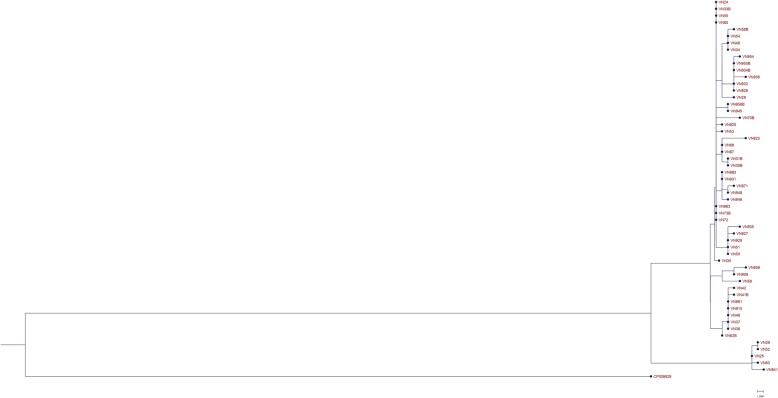
Single-nucleotide polymorphism analysis of 55 isolates of carbapenem-resistant *K. pneumoniae* isolated at a large Vietnamese hospital. Due to low average sequencing depth (<15x), samples VN26 and VN918 were excluded from the analysis. An ST15 *K. pneumoniae* (NCBI accession no: CP008929) isolated from blood at a Nepali hospital [16] was used as an outlier

The genetic relatedness of the isolates was determined with a SNP-analysis (Fig. 1). Due to low average sequencing depth (<15x), samples VN26 and VN918 were excluded from the analysis. The remaining 55 isolates formed two clusters consisting of 50 and 5 isolates respectively, with a minimum divergence between the clusters of 28 SNPs. The maximum divergence between the isolates in the larger clusters was 12 SNPs whereas the maximum divergence in the smaller cluster was 3 SNPs.

## Discussion

Characterization by using WGS data and antimicrobial susceptibility testing was carried out on 57 clinical isolates of CR-KP of ST15 collected at a Vietnamese pediatric hospital during 8 months in 2015 in order to characterize the strain in terms of antibiotic resistance, virulence and genetic relatedness. A shortcoming of the study is that the patient data were retrospectively collected and tracing the spread of ST15 via patients and staff was impossible to perform due to the unavailability of data.

The antibiotic resistance gene carriage varied between the isolates, likely due to carriage of different antibiotic resistance plasmids, although a few antibiotic resistance genes were shared amongst the majority of isolates, for example, *bla*_KPC-2_, *aac (6′)-*Ib and *dfrA23*. *bla*_KPC-2_ was the dominant carbapenemase gene and was carried by all isolates. Two isolates also carried *bla*_NDM-1_ in addition to *bla*_KPC-2_. Heterogeneity among antibiotic resistance genes and carbapenemase genes in particular are reflected in previous publications on ST15 outbreaks worldwide. Examples of reports include a clinical outbreak of ST15 *K. pneumoniae* with KPC-2 in Bulgaria [5], and NDM-1-producing ST15 in a Nepalese hospital outbreak [10]. In another ST15 outbreak at a hospital in Vietnam, the isolates carried predominantly *bla*_OXA-48_ (89%) but a few had *bla*_NDM-4_ (11%) [8]. The varied array of antibiotic resistance genes observed among the individual ST15 isolates in this study as well as in comparison with other studies on ST15 *K. pneumoniae* [5, 8, 10, 11] suggests a strain with a high capacity for horizontal gene transfer and, in particular, a propensity for acquisition of antibiotic resistance genes.

Susceptibility rates were highest for the antibiotics ceftazidime/avibactam (96%) and gentamicin (91%). Ceftazidime/avibactam is not approved for use in Vietnam and there are yet no international data supporting neonatology use. This indicates the urgent need, in the current epidemiological situation, for clinical trials evaluating treatment with antibiotic combinations as well as ceftazidime/avibactam in the patient group. However, two isolates carrying the carbapenemase gene *bla*_NDM-1_ showed high levels of resistance to ceftazidime/avibactam (MIC > 256 mg/L). Although none of the isolates in the study were susceptible to the aminoglycosides amikacin and tobramycin, likely due to the high rates of the aminoglycoside resistance genes *aac (6′)*-Ib and *aadA1*, all but five isolates were susceptible to gentamicin. These five gentamicin-resistant isolates carried the ribosomal methylase-gene *rmtB* and showed high levels of resistance to all tested aminoglycosides (MIC > 256 mg/L). While ceftazidime/avibactam and gentamicin showed the highest efficacy against the isolates in this study, overuse of either of these antibiotics may select for these corresponding resistance genes (i.e., *bla*_NDM-1_ and *rmtB*) and facilitate dissemination of the carrier genetic elements, further exacerbating the antibiotic resistance situation.

The rates of susceptibility to ciprofloxacin and chloramphenicol were very low, 0 and 3.5% respectively. Although chloramphenicol is not commonly used in Vietnam any longer, particularly among the type of patients included in the current study, fluoroquinolones are commonly used at Vietnamese ICUs [12]. The high resistance rate to ciprofloxacin (100%) emphasize the need for antimicrobial susceptibility testing to inform effective treatment. Trimethoprim/sulfamethoxazole and fosfomycin susceptibilities were 70% to both antibiotics. For trimethoprim/sulfamethoxazole, 17 isolates were resistant. High levels of resistance (MIC > 32 mg/L) was displayed by eight isolates, all of which carried *sul1* and *sul2* (sulphonamide resistance genes), and *dfrA16* and *dfrA23* (trimethoprim resistance genes).

The colistin resistance rate among the studied ST15 isolates was high (42%) and is a cause of major concern since colistin combination treatment is the most effective treatment option against CR-KP infections available today in Vietnam. The colistin resistance mechanisms of the 21 colistin-resistant isolates have been reported on elsewhere [14]. The resistance phenotype in these isolates could be attributed to chromosomal changes in *mgrB*, a known loss-of-function colistin resistance gene. Among these isolates, seven different genetic events in *mgrB* predicted to lead to disrupted functionality of the gene product were observed. The high genetic relatedness of the colistin-susceptible and colistin-resistant ST15 isolates combined with the high heterogeneity in mutations leading to resistance in the resistant isolates, are indicative of a strain exposed to selection pressure via colistin, possibly in the hospital environment. None of the plasmid-mediated colistin resistance genes of the *mcr* family could be detected among the ST15 isolates in this study, nevertheless, *mcr-1* has previously been reported in three isolates of CR-KP of ST307 at the study hospital [17]. Continued resistance-monitoring and prudent use of colistin are imperative to prevent dissemination of *mcr-1* and further emergence and selection of colistin resistance-engendering mutations in this hospital setting.

All isolates were determined to carry the *mrkABCDFJ* operon encoding the type 3 fimbriae system, a virulence factor that is associated with biofilm formation and enhanced capacity for binding to abiotic surfaces, which could enhance the survival of the strain in hospital environments and increase its capacity for invasiveness via for example endotracheal tubes and catheters. 84% of the isolates carried the operon encoding the aerobactin siderophore. Production of aerobactin is overrepresented among hypervirulent strains of *K. pneumoniae*, however, the siderophore does not appear to be a factor by itself sufficient to engender the hypervirulence phenotype and the operon is also present among non-hypervirulent strains albeit at lower ratios [18].. One isolate carried *rmpA2*, a gene associated to hypervirulence. However, mutations in this gene resulted in a heavily truncated, and likely dysfunctional protein. The capsular serotype was determined to be K10 for 55 isolates, a serotype not typically associated to hypervirulence. Serotypes of the remaining two isolates (VN915B and VN918) were indeterminable, likely due to assembly or sequencing problems. Given the data presented above, it is unlikely that any of the isolates analyzed in this study could be considered hypervirulent. Most of the isolates were from tracheal fluid or nasopharynx (84%), which could indicate colonization rather than infection. The mortality rate was 14%, however, 19 patients were withdrawn from medical treatment due to pessimal prognosis. Although follow-up information for these patients were not available, the outcome for withdrawn patients is usually death, meaning the actual mortality rate likely lie in the range of 14–48%. The patient group studied had high rates of co-morbidities, and colonization with CR-KP may not have been a factor in the patient outcome in several cases. Nevertheless, colonization with carbapenem-resistant Enterobacteriales has been shown to increase the risk of subsequent infection with the pathogen [19], and the isolates in the current study were collected from ICUs and neonatal wards, where the patients are likely to be immunologically vulnerable. This may enhance the capacity of this strain to be invasive, as also indicated by the fact that four isolates were recovered from blood.

The SNP-analysis showed that the isolates were closely related genetically, but clustered into two sub clusters (Fig. 1) constituted of 50 and 5 isolates respectively separated by 28 SNPs. The low maximum divergence within each cluster were, 12 and 3 SNPs respectively, indicate that these clusters constitute two clones [20]. The five isolates constituting the smaller cluster were homogenous in terms of antibiotic susceptibilities, the exception being VN941 which was resistant to colistin (MIC = 16 mg/L). The colistin resistance in this isolate was due to an insertion sequence transposition into the PhoP/PhoQ two component system regulatory gene *mgrB* as previously reported [14]. Antibiotic resistance gene carriage in the isolates were also similar, except for VN941 which alone in the cluster carried *aac (6′)*-Ib and lacked *bla*_TEM-1B_. The isolates in this cluster carried the same antibiotic resistance genes and were notably the only isolates in the study which carried the aminoglycoside resistance gene *rmtB* and were resistant to gentamicin. However, there was a high diversity in terms of antibiotic susceptibility and carriage of antibiotic resistance genes in the isolates within the clusters. This indicates that the clones might have disseminated throughout the hospital and over time taken up plasmids containing antibiotic resistance genes. Strains of ST15 have previously been shown to have a high propensity for dissemination and circulation in hospital environments and to cause hospital-acquired infections, as reported in hospital outbreaks in the Netherlands [11] and in Nepal [16]. A large proportion of the patients from which the isolates were collected (78% of the patients for which data was available) were transferred from other hospitals, and so another possibility would be that this ST15 strain is well-established in many different hospital settings in Vietnam and the difference in resistance gene and plasmid carriage reflects the plasmid diversity in the local settings. Regardless of origin, there is an urgent need for infection controls measures and antibiotic stewardship to minimize transmission of and selection for this and other carbapenem-resistant strains.

## Conclusions

In the current study, multidrug-resistant CR-KP belonging to the ST15 strain from a large Vietnamese pediatric hospital were characterized with WGS and antimicrobial susceptibility testing. The diverse variety of antibiotic resistance genes among the isolates and the high antibiotic resistance rate is indicative of a highly adaptable strain. This emphasizes the importance of implementation of infection control measures, continued monitoring of antibiotic resistance and prudent use of antibiotics to prevent further selection of resistant strains and the emergence of pan-resistant clones.

## Data Availability

WGS data generated/analyzed in this study is available at the Sequence Read Archive at NCBI (https://www.ncbi.nlm.nih.gov/sra) with the accession numbers SRR6208298-SRR6208328 and SRR6656601-SRR6656635.

## Abbreviations

aa: Amino acid
CR-KP: Carbapenem-resistant *Klebsiella pneumoniae*
ICU: Intensive care unit
SNP: Single-nucleotide polymorphism
WGS: Whole-genome sequencing
